# Surface Proteomic Analysis Reveals the Presence of Noncanonical Cell Membrane Endoplasmic Reticulum Chaperones in High-Grade Gliomas

**DOI:** 10.1021/acs.jproteome.5c00616

**Published:** 2025-11-25

**Authors:** Alexis Z Minchaca, Jean Bertoldo, Philipp Graber, Dong-Hun Bae, Nisitha Jayatilleke, Chelsea Mayoh, Brett W. Stringer, Louise Ludlow, Maria Kavallaris, Angelica M. Merlot

**Affiliations:** † Children’s Cancer Institute Australia, Lowy Cancer Research Centre, UNSW, Sydney, NSW 2052, Australia; ‡ School of Clinical Medicine, Faculty of Medicine and Health, UNSW, Sydney, NSW 2052, Australia; § UNSW Centre for Childhood Cancer Research, Faculty of Medicine & Health, 7800 University of New South Wales, Kensington, NSW 2031, Australia; ∥ UNSW Australian Centre of Nanomedicine, Faculty of Engineering, UNSW, Sydney, NSW 2052, Australia; ⊥ UNSW RNA Institute, Faculty of Science, UNSW, Sydney, NSW 2052, Australia; # Institute for Biomedicine and Glycomics, 5723 Griffith University, Brisbane, QLD 4111, Australia; ∇ Murdoch Children’s Research Institute, Parkville, VIC 3053, Australia; ○ Children’s Cancer Centre, The Royal Children’s Hospital, Parkville, VIC 3053, Australia; ◆ Department of Paediatrics, The University of Melbourne, Parkville, VIC 3052, Australia

**Keywords:** High-grade glioma, surfaceome, membrane proteins, surface expression, endoplasmic reticulum chaperones

## Abstract

High-grade gliomas (HGG) are highly aggressive tumors, which are predominately fatal for adults and pediatric patients. Identifying cancer-selective therapeutic targets remains a critical unmet need. The overexpression of endoplasmic reticulum (ER) chaperones in various cancers is well documented. Moreover, tumor cells exhibit an atypical surface expression of ER chaperones, suggesting the potential for selective targeting. Our study examined the differences in the mRNA, total protein, and surface expression levels of seven key ER chaperones, compared with those in non-neoplastic samples. Notably, a poor correlation was found between mRNA, protein, and surface protein levels, underscoring the limitations of transcriptomics alone in target discovery. We also highlight the limitations of surfaceome studies which exclude noncanonical membrane proteins, such as ectopically expressed ER chaperones, which often escape detection by conventional bioinformatic pipelines. For the first time, this study advances our understanding of the surface expression of ER chaperones in both adult and pediatric HGG. Our findings highlight the importance of surfaceome analysis in the discovery of cancer selective targets against this devastating disease.

## Graphical abstract

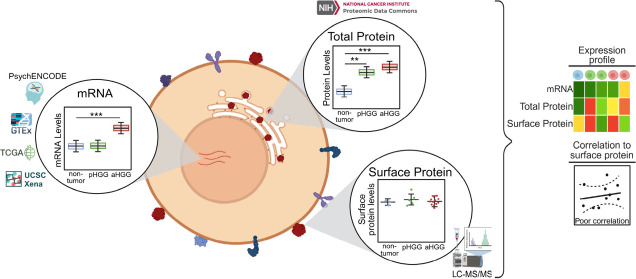

## 1 Introduction

High-grade gliomas (HGG) are the most common central nervous system (CNS) tumors.^1^ Among adult HGG (aHGG), glioblastoma IDH-wildtype (formerly glioblastoma; referred to as aHGG from herein) is the most common malignant brain cancer in adults accounting for 50% of the malignant CNS tumors, with a calculated overall survival (OS) of around 15 months.^2^ Pediatric HGGs (pHGG) are less frequent, but just as devastating, with an overall survival (OS) of 10–73 months.^2^ Despite key differences between aHGG and pHGG (accurately reflected in the 2021 WHO Classification of Tumors of the Central Nervous System (CNS) or WHO CNS 5), the same therapeutic strategy (including surgical resection, radiotherapy and chemotherapy with Temozolomide), which has not changed in recent decades, is currently utilized with limited efficacy.^2^

Elucidating new selective therapeutics is critical for the battle against this incurable disease. In this context, members of the endoplasmic reticulum (ER) chaperone family have been of interest for their differential expression in different cancers.^3,4^ Elevated ER chaperone expression is a well-established marker of the unfolded protein response (UPR), a stress-adaptive pathway activated by microenvironmental challenges, including those present in the tumor microenvironment, such as hypoxia, nutrient deprivation, and therapy-induced stress.^5^ A higher expression of the ER chaperones in tumors compared to nonmalignant cells is a well-described adaptive ER stress response, favoring tumoral cell survival.^6–9^ Moreover, the overexpression of ER chaperones in cancer cells (*e.g.*, colon, gastric, or myeloproliferative diseases) has been associated with their translocation to atypical cell compartments, such as the cell surface.^10^ CALR, CANX, GRP78, GRP94, GRP170, HSP47, and members of the PDI family are some of the chaperones that have been identified at the cell surface in different cancer types (*e.g.*, neuroblastoma, lung adenocarcinoma, ovarian cancer, *etc.*).^3,4,11–16^ ER chaperones translocate to the cell surface due to ER retention escape, vesicle or exosome trafficking, or unconventional secretion, triggered by ER stress, oncogenic signaling or therapy-induced stress.^10,17,18^ ER chaperone translocation can occur in non-neoplastic cells, but it is typically transient and linked to regulated stress responses or immune signaling.^10^ In cancer, this process is more amplified and frequently rewired for tumor-promoting roles.^19^ The increased expression and atypical surface location of ER chaperones has increased efforts to investigate their potential as anticancer targets with promising outcomes observed *in vitro* ^14,15,20,21^ and *in vivo*.^22,23^ However, a complete expression profile of these proteins in adult and pediatric HGG, especially ER chaperone surface expression, has not been reported. Taking this into consideration, we aimed to characterize the ER chaperone landscape in aHGG and pHGG from a multiomics perspective, using clinically relevant adult and pediatric samples. Herein, we evaluated the mRNA and protein expression levels in HGG, low-grade glioma (LGG) and non-neoplastic brain (NNB) tissues. Moreover, we implemented a mass spectrometry (MS)-based analysis of the surfaceome of various cell lines, patient-derived cells and NNB cells, to assess ER chaperone surface translocation for the first time in both adult and pediatric HGG models.

The discovery of new surface targets has predominantly relied on genomic and transcriptomic data, although there is often a lack of correlation of mRNA levels with protein expression.^24,25^ Here, we examined the differences between mRNA, total protein, and surface expression levels of seven ER chaperones in HGG. Our analysis highlights the poor correlation and significance of integrating gene and protein expression, including the subcellular localization (*e.g.*, cell surface) of proteins, to better exploit the unique vulnerabilities of cancer cells.

## 2 Experimental Procedures

### 2.1 Gene Expression Analysis

For adult patient samples, RNA-seq data corresponding to *CALR*, *CANX*, *HSPA5*, *HSP90B1*, *HYOU1*, *P4HB*, and *SERPINH1* genes in LGG (*n* = 344) and HGG (*n* = 221) were obtained from the Cancer Genome Atlas (TGCA) and reclassified based on the WHO CNS5 2021 classification.^26,27^ NNB samples (*n* = 1142–1148) were included from the GTEx study through the UCSC XENA browser.^27^ RNA-seq data were processed using the UCSC XENA browser.^27^ For the pediatric cohort, we obtained the NNB sample data (*n* = 34) from the PsychENCODE Consortium (http://development.psychencode.org/).^28^ The pLGG (*n* = 22) and pHGG (*n* = 75) samples were obtained from the St Jude Cloud.^29^ Violin plots and statistical analysis were performed in GraphPad Prism (v10.2.1). For the pHGG data, principal component analysis (PCA) was conducted to distinguish tumor from non-neoplastic tissues using the prcomp function in base R with default configuration.^30^ To evaluate potential technical batch effects before performing differential expression analysis, PCA was also applied to a subset of housekeeping genes obtained from the Housekeeping and Reference Transcript Atlas using the prcomp algorithm.^31^ The absence of batch effects was confirmed through a visual assessment of the PCA plots. Differential expression analysis was subsequently performed using the DESeq2 package in R (v4.2.3).^32^ For the adult HGG cell lines, QIMR normalized RNA-seq data were obtained from the Q-Cell Web site (https://www.qimrb.edu.au/qcell).^33–35^

### 2.2 Survival Analysis

Patient overall survival (OS) was evaluated relative to high and low mRNA expression defined by the first (Q1) and last (Q4) quartile, using the R2: Genomic analysis and visualization platform (http://r2.amc.nl). The “TCGA-540-MAS5.0-u133a” study (*n* = 540), and the “Tumor Glioma Pediatric-Paugh-53-MAS5.0-u133p2” study (*n* = 53)^36^ were chosen for the analysis of adult and pediatric data, respectively. Results were visualized as Kaplan–Meier curves.

### 2.3 Genomic Alterations Analysis

Genomic alterations were visualized using the oncoplot tool in the cBioPortal database (https://www.cbioportal.org/).^37,38^ Adult HGG data (*n* = 221) were obtained from the TCGA study (reclassified as described in section 2.1). Pediatric HGG data (*n* = 22) were obtained from the Clinical Proteomic Tumor Analysis Consortium (CPTAC).^39^

### 2.4 Proteomic Analysis

The “Pediatric Brain Cancer Pilot Study” (PDC000180)^39^ and the “CPTAC GBM Discovery Study” (PDC000204)^40^ data were used to generate pHGG and aHGG ER chaperone protein expression heatmaps, respectively, using the protein quantitation tool from the Protein Data Commons server.^41^ Expression analysis within the data sets for each individual chaperone in aHGG (*n* = 99) vs. NNB (*n* = 10) or pHGG (*n* = 22) vs. pLGG (*n* = 86) was performed using the UACLAN server (http://ualcan.path.uab.edu/index.html).^42^ These data sets possessed a robust number of samples, although it did not include aLGG or pediatric NNB. Hence, the smaller proteomic data set PXD015545 (accessed through the ProteomeXchange Consortium)^43^ was used to investigate the proteomic expression of ER chaperones in aHGG IDH wildtype (*n* = 39), aLGG (*n* = 9), and NNB (*n* = 4). The published MS-quantified and normalized data and patient metadata were downloaded for analysis.^43^ For the adult HGG cell lines, QIMR log_2_-normalized proteomic data were obtained from the Q-Cell Web site (https://www.qimrb.edu.au/qcell).^33–35^ GraphPad Prism (version 10.2.1) was used for statistical analysis and visual representation.

### 2.5 Transmembrane Domain and Glycosylphosphatidylinositol (GPI) Anchoring Prediction

The ER chaperone amino acid sequence was obtained from UniProt (using the accession numbers specified in Table 1). Deep TMHMM 1.0 or NetGPI 1.1 (Technical University of Denmark (DTU), Denmark) were used for transmembrane domain or GPI anchoring prediction.^44,45^

**Table 1:** Endoplasmic Reticulum Chaperones Investigated in High-Grade Glioma (HGG) Samples

UniProt Accession Number	Gene Symbol	Protein Symbol^a^	Common Protein Name(s)
**P11021**	*HSPA5*	**GRP78**	Heat shock protein family A member 5, Binding Immunoglobulin Protein (BiP), or 78 kDa glucose-regulated protein
**P14625**	*HSP90B1*	**GRP94**	94 kDa Glucose-regulated protein
**P27797**	*CALR*	**CALR**	Calreticulin
**P27824**	*CANX*	**CANX**	Calnexin
**P07237**	*P4HB*	PDI/**PDIA1**	Protein disulfide-isomerase
**Q9Y4l1**	*HYOU1*	**GRP170**/ORP150	150 kDa oxygen-regulated protein or 170 kDa Glucose regulated protein
**P50454**	*SERPINH1*	**HSP47**	47 kDa heat shock protein

a For consistency, the preferred name used herein for each protein is indicated in bold.

### 2.6 Ethics Approval

The obtention of adult and pediatric patient-derived HGG cells was approved by the University of New South Wales Human Research Ethics Committee under ID numbers HC210029, iRECS6940 and HC210292.

### 2.7 Cell Lines and Culture Conditions

Cells were cultured at 37 °C in a humidified atmosphere of 5% CO_2_ (Binder, Germany). The aHGG cell line T98G (ATCC CRL-1690) was purchased from the American Type Culture Collection (ATCC, USA) and cultured in DMEM medium (Thermo Fisher Scientific, Australia) supplemented with 10% FBS (Thermo Fisher Scientific), and 1% l-Glutamine (Merck, Australia). For the NNB models, cerebral microvascular endothelial cells (HBEC-5i) were purchased from ATCC and grown in DMEM/F12 media (Thermo Fisher Scientific) with endothelial growth supplement (Merck) and 10% FBS (Thermo Fisher Scientific) in 0.1% gelatin-coated flasks (Assay Matrix, Australia). Human brain vascular pericytes (HBVP) were purchased from ScienCell Research Laboratories (USA) and were grown in Pericyte Medium supplemented with Pericyte Growth Supplement and 10% FBS (all reagents from ScienCell Research Laboratories). Normal human astrocytes (NHA-SV40) were purchased from Creative Bioarray (USA) and grown using the Astrocyte Growth Medium (AGM) BulletKit (Lonza, Switzerland). HBVP and NHA-SV40 cells were grown in poly-l-Lysine (Australian Bioresearch, AU) coated T75 cm^2^ flasks (Corning, USA).

A collection of 11 aHGG () patient derived cells (PDC) was obtained from QIMR Berghofer Medical Research Institute (Queensland, Australia).^33–35^ These cells were grown in StemPro Neural Stem Cell Serum-Free (NCS SFM) cell culture media (Thermo Fisher Scientific) in 1% Matrigel coated T75 cm^2^ flasks (Corning). Pediatric HGG samples () were obtained from ZERO^46^ (*n* = 3, referred to as zccs116, zccs414 and zccs231) and Children’s Cancer Centre (CCC; *n* = 2, pHGG04-05) Australian biobanks and cultured as recommended.^47^ Briefly, zccs116, zccs414 and zccs231 from ZERO were cultured as neurospheres in T75 cm^2^ flasks (vertical position), grown in Neurobasal-A Medium - DMEM/F12 (50/50% v/v) supplemented with HEPES, sodium pyruvate, MEM nonessential amino acids GlutaMAX-I and B27 supplement (without vitamin A); all from Thermo Fisher Scientific, hEGF and hFGF-basic (154aa) growth factors from Jomar Life Research (Australia), and 0.2% heparin solution from StemCell Technologies (Canada). Single cell suspensions were obtained by incubation with Accutase (Sigma-Aldrich) and mechanical dissociation. Samples pHGG04 and pHGG05 from CCC were obtained directly from the patient’s tumor and lacked expansion potential. Therefore, the samples were thawed and recovered (for approximately 4 h) in a 6-well ultralow adherent plate containing DMEM/F12 media supplemented with Glucose Solution, N2 supplement, and B27 supplement (all from Thermo Fisher Scientific), hEGF and hFGF-basic (154aa) growth factors from Jomar Life Research, 0.2% heparin solution from StemCell Technologies, and gentamicin from Merck (Australia).

### 2.8 Cell Surface Protein Biotinylation and Neutravidin Pulldown

Cells were grown in 10 cm dishes (Corning) to 80% confluency, washed twice in ice-cold PBS and incubated in ice-cold EZ-Link Sulfo-NHS-SS-biotin (0.5 mg/mL in PBS; Thermo Fisher Scientific), with gentle rocking at 4 °C for 30 min for cell surface protein labeling.^4^ The biotinylation reaction was quenched with 3–5 mL of ice-cold 100 mM glycine in PBS, pH 7.4. Lastly, cells were washed with ice-cold PBS and lysed using high-salt radioimmune precipitation (RIPA) buffer (500 mM NaCl) in the presence of 1% protease inhibitors (Roche, Switzerland) and 10% phosphatase inhibitors. Protein concentration was determined by the bicinchoninic acid method (BCA protein assay kit, Thermo Fischer Scientific).^48^ To isolate the surface protein content, 0.1–0.3 mg of total protein were mixed with Pierce High Capacity Neutravidin agarose beaded resin (Thermo Fisher Scientific) with end-to-end rotation for 1 h at room temperature. The resin was washed with a high-salt RIPA buffer 10 times, and the bound proteins were eluted in the presence of 50 μL of a 20 mM HEPES buffer with 100 μM DTT (pH 7.4) for 20 min at room temperature.

### 2.9 LC-MS/MS Analysis

Samples were prepared and analyzed at the BMSF within the Mark Wainwright Analytical Centre (UNSW, Sydney). Briefly, samples were reduced, alkylated, and digested with 100 ng of sequencing grade modified trypsin (Promega, Australia) at 37 °C, overnight. The digested peptides were separated by nanoLC (Ultimate nanoRSLC UPLC and autosampler system; Dionex, The Netherlands). Samples (2.5 μL) were concentrated and desalted with a micro C18 precolumn (300 μm × 5 mm, Dionex) with H_2_O:CH_3_CN (98:2, 0.1% Trifluoroacetic acid; Honeywell Research Chemicals, USA) at 15 μL/min. After a 4 min wash, the precolumn was switched (Valco 10 port UPLC valve, Valco, USA) into line with a fritless nano column (75 μm × ∼25 cm) containing C18AQ media (1.9 μm, 120 Å Dr Maisch, Ammerbuch-Entringen, Germany). Peptides were eluted using a linear gradient of H_2_O:CH_3_CN (98:2, 0.1% formic acid; Univar, Australia) to H_2_O:CH_3_CN (64:36, 0.1% formic acid; Univar) at 200 nL/min over 30 min. High voltage (2000 V) was applied to low-volume Titanium union (Valco), and the tip was positioned ∼0.5 cm from the heated capillary (275 °C) of an Orbitrap Fusion Lumos (Thermo Electron, Germany) mass spectrometer (data-dependent acquisition (DDA) mode). Positive ions were generated by electrospray.

### 2.10 Protein Identification and Label-Free Quantification

Peak lists were generated using the Mascot Daemon/Mascot Distiller (Matrix Science, England)^49^ to produce ProteomeXchange files. Label-free quantification (LFQ) was performed in MaxQuant (v2.1.3.0). Database search was conducted with the integrated Andromeda search engine against the Human Reference Proteome (UniProt proteome ID: UP000005640, 20,586 entries).^50,51^ Carbamidomethylation of cysteines were set as fixed modification, and methionine oxidation and N-terminal acetylation as variable modifications. Enzyme specificity was set to trypsin with up to two missed cleavage sites. Precursor mass tolerance was 4.5 ppm, and peptide fragment was 0.5 Da. All peptides were used in the quantification, with the match between runs enabled. The false discovery rate (FRD) was controlled using a target-decoy approach, with both protein and peptide FDR thresholds set at 1%. LFQ data were processed in Perseus software (version 2.0.7.0, Max Plank Institute of Biochemistry).^52^ Proteins were filtered for contaminants, decoys and peptides identified only by site.^52^ Sample quality was determined based on 2D-Principal Component Analysis and correlation within groups. LFQ values were transformed to Log_2_(LFQ) for analysis. Sample groups were defined by categorical annotation into non-neoplastic brain (NNB) and HGG. Values were present in at least 50% of the samples for each group (NNB and HGG), with 4 or more identified peptides. The presence of the 7 ER chaperones of interest was investigated within the identified proteins. The Log_2_(LFQ) processed data for the ER chaperone identifications, as well as each identified peptide are presented in , respectively. The MS raw data have been deposited to the ProteomeXchange Consortium *via* the PRIDE^53^ partner repository with the data set identifier PXD061211.

### 2.11 Correlations

Pearson correlation coefficients were calculated between surface protein and mRNA or total protein expression for each chaperone across the 11 QIMR cell lines.^33–35^ The mRNA and total protein expression was obtained from Q-cell (https://www.qimrb.edu.au/qcell).^33–35^ For each ER chaperone, the mRNA and total protein data from each replicate (*n* = 3) were averaged and converted to z-score values. These data were correlated to the z-score value for the surface expression of each chaperone obtained herein across the 11 cell lines. These z-score values were used to build the heatmap of expression across the different cell lines for each chaperone at the mRNA, total protein, and surface protein levels.

### 2.12 Statistical Analysis

For the mRNA expression, Brown-Forsythe and Welch ANOVA test with Games-Howell’s multiple comparison test was used to compare each group to the non-neoplastic data set. Wilcoxon test or chi-square test analysis were performed in HGG samples to compare wildtype and altered (mutated) ER chaperone mRNA in relation to age, sex, and radiotherapy treatment. Surfaceome data generated in this study was analyzed using a Kruskal–Wallis test with Dunn’s posthoc test for multiple comparisons. Proteomic data from the public data set PXD015545 ^43^ was analyzed using an unpaired *t*-test for each ER chaperone evaluated. Where pertinent, normality assumptions were checked with Q-Q and residual plots. Pearson correlation, *p* value, and 95% confidence intervals were calculated using GraphPad Prism (v10.2.1).

### 2.13 Experimental Design

The specific data sets used for the analysis of mRNA expression, genomic alterations, survival analysis, and total protein expression are provided in each figure legend. When pertinent, the online server used to analyze the data and generate the figures is also indicated. For the surface proteome evaluation, a collection of 11 aHGG PDC, and 5 pHGG PDC were obtained. Given the lack of expansion capacity or the limited material of the patient-derived pediatric samples, the surfaceome of PDCs was evaluated as one replicate. The NHA-SV40, hBEC-5i and HBVP surfaceome were evaluated as biological replicates (*n* = 3) and the log_2_ LFQ intensity values were averaged, with the mean value used for statistical comparison. Samples were grouped into NNB (*n* = 3), pHGG (*n* = 5), and aHGG (*n* = 11). The nonparametric Kruskal–Wallis test, followed by Dunn’s post-test was used to compare surface ER chaperone protein expression given the sample size of the first group, where a robust assessment of equal variance and normality was limited.

## 3 Results

### 3.1 ER Chaperone Gene Expression Is Altered in Adult and Pediatric HGG

Among the ER chaperone family, CALR, CANX, GRP78, GRP94, GRP170, HSP47, and PDIA1 (Table 1) have been detected at the surface of cancer cells (reviewed in refs 19, 54, 55 ). GRP78, GRP94, CALR, and PDIA1 represent some of the most abundant ER chaperones (key players in protein folding and ER stress signaling pathways) and are the most studied tumor surface targets.^55–59^ In contrast, less abundant but functionally significant members, such as GRP170 and HSP47, have been less studied as surface proteins but are frequently upregulated in cancer and play key roles in tumor angiogenesis and progression, respectively.^10,55^ Therefore, using publicly available data sets, we evaluated the mRNA expression of these 7 ER chaperone genes in aHGG and pHGG tumor samples, compared to low-grade glioma (LGG), and non-neoplastic brain (NNB) tissue, to investigate their association with tumor grade. Interestingly, a significant (*p* < 0.0001) upregulation of the mRNA of all 7 ER chaperones was observed in aHGG (*n* = 221) compared to aLGG (*n* = 344), and to non-neoplastic brain tissue (*n* = 1142–1148; Figure 1A–G). Similarly, ER chaperone expression was increased with the tumor diagnosis and grade (NNB < LGG < HGG; *p* < 0.0001; Figure 1A–G). On the other hand, the mRNA expression profile varied among the pediatric tumor cohort (pLGG *n* = 22; pHGG *n* = 75) and the pediatric NNB (*n* = 34; Figure 1H–N). A significant overexpression of *HSP90B1* (*p* < 0.01), and *CANX* (*p* < 0.0001) in pHGG compared to NNB was determined, while *HSPA5*, *P4HB*, *HYOU1*, and *SERPINH1* remained unchanged (Figure 1H,I,K–N). In contrast with the observation on aHGG data, *CALR* was found significantly (*p* < 0.05) reduced in pHGG in relation to the noncancerous samples (Figure 1J). Similarly, *CANX* was the only ER chaperone mRNA found to be upregulated (*p* < 0.01) in pLGG compared to NNB (Figure 1K) while *CALR*, *P4HB*, and *SERPINH1* showed a significantly (*p* < 0.01–0.05) lower expression (Figure 1J,L,N) and the rest remained unchanged. Moreover, no clear associations with expression or tumor grade were observed based on the expression levels of any of the chaperones evaluated in pHGG (Figure 1H–N).

**Figure 1:**
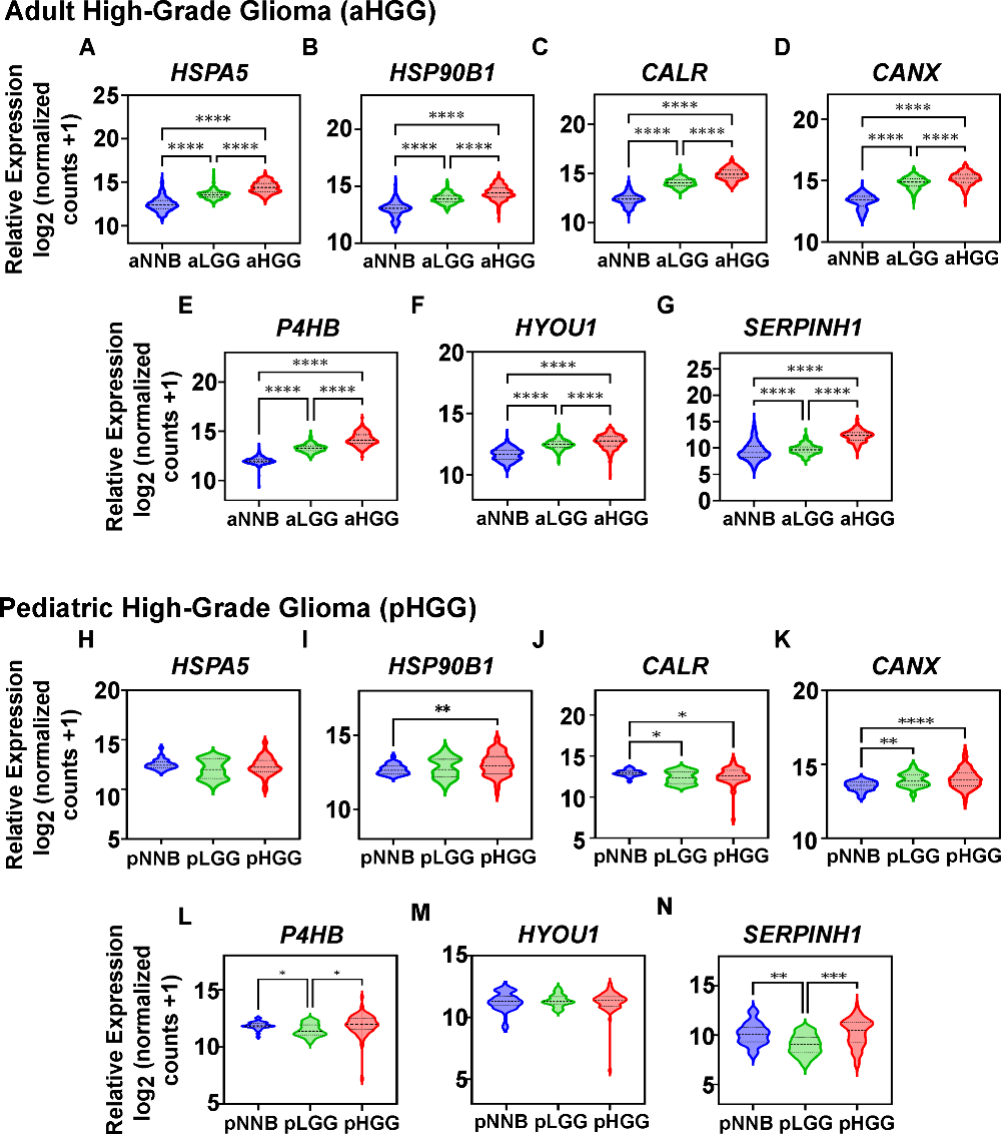
ER chaperones gene expression in adult and pediatric HGG and LGG patients. The mRNA expression of the ER chaperones, (A) *HSPA5*, (B) *HSP90B1*, (C) *CALR*, (D) *CANX*, (E) *P4HB*, (F), *HYOU1* and (G) *SERPINH1*, was assessed in adult non-neoplastic brain tissue (aNNB; *n* = 1142–1148; represented in blue), adult low-grade glioma (aLGG; *n* = 344; represented in green), and adult high-grade glioma (aHGG; *n* = 221; represented in red) tissue samples. Data from the TCGA and GTEX studies (TCGA-TARGET-GTEX data set) were analyzed through the Xena Browser (UCSC).^27^ The samples were reclassified according to the latest WHO CNS5 classification (based on ref 26). Similarly, the expression of (H) *HSPA5*, (I) *HSP90B1*, (J) *CALR*, (K) *CANX*, (L) *P4HB*, (M), *HYOU1*, and (N) *SERPINH1* in pediatric NNB (pNNB; *n* = 34; represented in blue), obtained from PsychENCODE,^28^ was compared to pediatric LGG (pLGG; *n* = 22; represented in green) and pediatric HGG (pHGG; *n* = 75; represented in red), obtained from St. Jude Cloud.^29^ One-way ANOVA with Games-Howell’s multiple comparison test was performed to compare groups using GraphPad Prism (v10.2.1). Median (thick line), first and last quartiles (thin lines) are represented in the violin plots. *p < 0.05, **p < 0.01, ****p < 0.0001. aHGG: adult high-grade glioma; pHGG: pediatric high-grade glioma.

The presence of ER chaperone variants has been described in certain cancers including myeloproliferative neoplasms and gastric cancer.^23,60,61^ The presence of tumor-specific mutated proteins offers an advantage in terms of therapeutic cancer selectivity. Therefore, we investigated the presence of genomic alterations (missense, splice, and truncation mutations, structural variants, amplifications, and deletions) across the ER chaperones of interest. The aHGG cohort included 0–2.3% of samples whose ER chaperone was altered, with *HYOU1* being the most frequently mutated gene (), with no significant correlation with clinical data (*age*, sex, or radiotherapy; ). In contrast, the pHGG cohort showed a single sample (5%) with *P4HB* amplification (). These results are consistent with the low mutational burden characteristic of brain tumors.^39^

### 3.2 ER Chaperone Expression Levels Can Be of Prognostic Value for Adult HGG Patients

In the adult cohort, higher levels of ER chaperone mRNA were observed in the most aggressive glioma samples analyzed (HGG) compared to the less aggressive form (LGG; Figure 1) and non-neoplastic samples (NNB; Figure 1). Given these findings, patient overall survival (OS) in aHGG and pHGG cohorts was analyzed comparing the last and first quartile (high and low expression, respectively; aHGG *n* = 254 and pHGG *n* = 26) for each ER chaperone independently. The Kaplan–Meier analysis showed that a high expression (red line) of *HSPA5*, *HSP90B1*, *P4HB*, or *SERPINH1* (*p* < 0.0001–0.05) was associated with a poorer OS in aHGG patients compared to the low expression cohort (blue line; Figure 2A,B,E,G). However, no significant change in OS was observed with high or low expression levels of the remaining 3 chaperones, namely, *CALR*, *CANX and HYOU1* (Figure 2C,D,F). In contrast, no association with OS was found in the pHGG samples investigated (Figure 2H–N).

**Figure 2:**
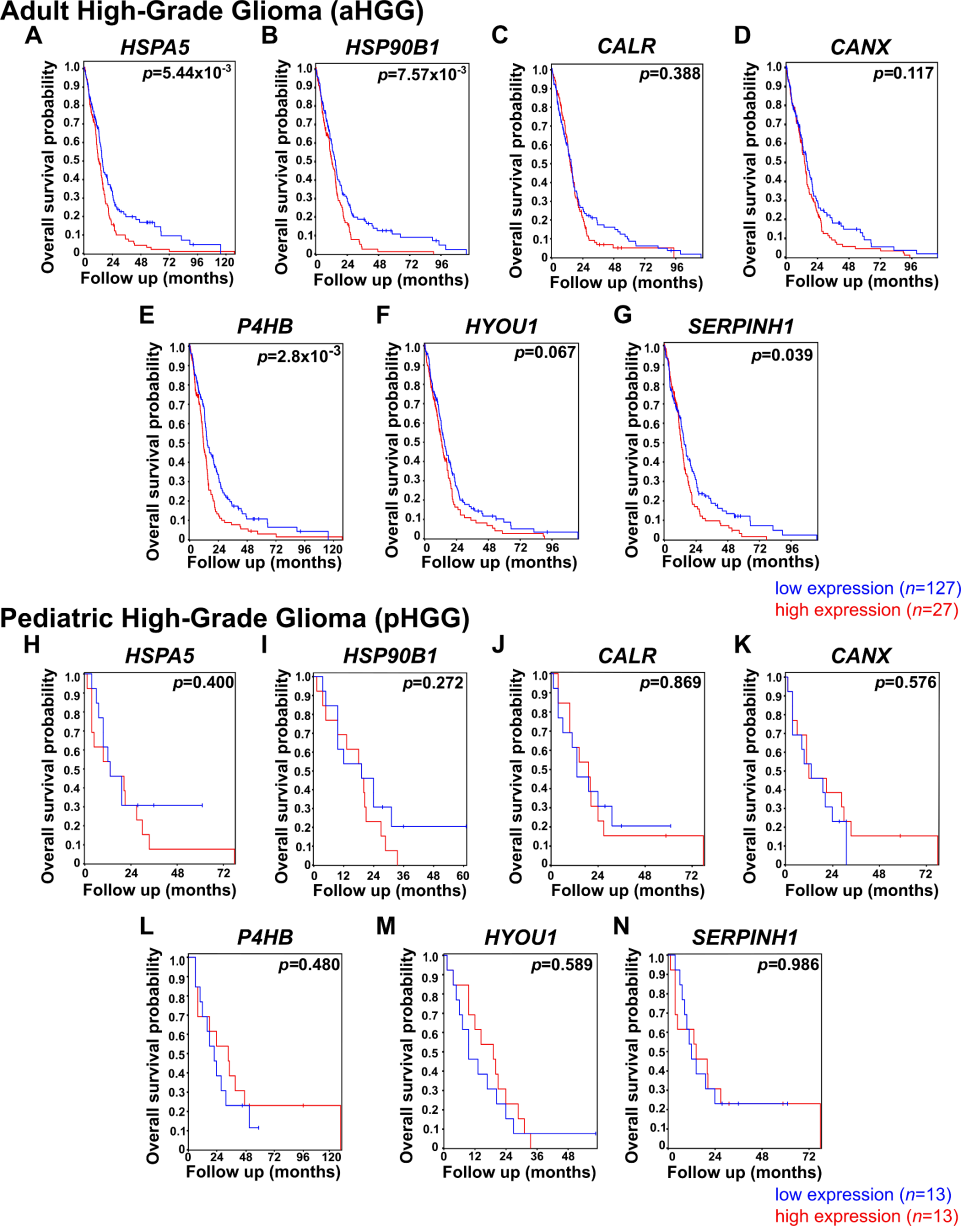
ER chaperone mRNA overexpression of *HSPA5*, *HSP90B1*, *P4HB*, and *SERPINH1* was associated with a poorer prognosis in adult HGG patients. Overall survival in adult HGG (aHGG; TCGA-540-MAS5.0-u133a study) and pediatric HGG (pHGG; Paugh-53-MAS5.0-u133a study)^36^ patient samples was compared between low (first quartile, blue line) and high (last quartile, red line) mRNA expression samples (aHGG *n* = 127, pHGG *n* = 13, per group) for each ER chaperone. Data and graphics were processed and downloaded from R2 genomic analysis and visualization platform (https://hgserver1.amc.nl/cgi-bin/r2/main.cgi). Statistical significance was calculated by using a log-rank test.

### 3.3 The Majority of ER Chaperone Proteins Are Overexpressed in HGG Compared to LGG or Normal Tissue Samples

As previously described, there is a low association between mRNA abundance and protein expression levels.^62^ Therefore, we investigated the protein levels of seven ER chaperones in HGG using publicly available mass spectrometry (MS)-based proteomes derived from clinical samples. A significant overexpression (*p* < 0.0001) of ER chaperones was observed in aHGG (*n* = 99) compared to aNNB (*n* = 10) except for GRP170 (Figure 3A–G) in the “Clinical Proteomic Tumor Analysis Consortium (CPTAC) GBM Discovery Study” data set.^40^ Similar results were observed for aHHG (*n* = 39) compared to aLGG (*n* = 9; ; ProteomeXChange Data set Identifier: PXD015545). Additionally, the heatmap (Figure 3H) showed an overall pattern in which a high relative expression (z-score) of one of the chaperones within a sample (each column) is associated with the elevated relative expression of the other chaperones.

**Figure 3:**
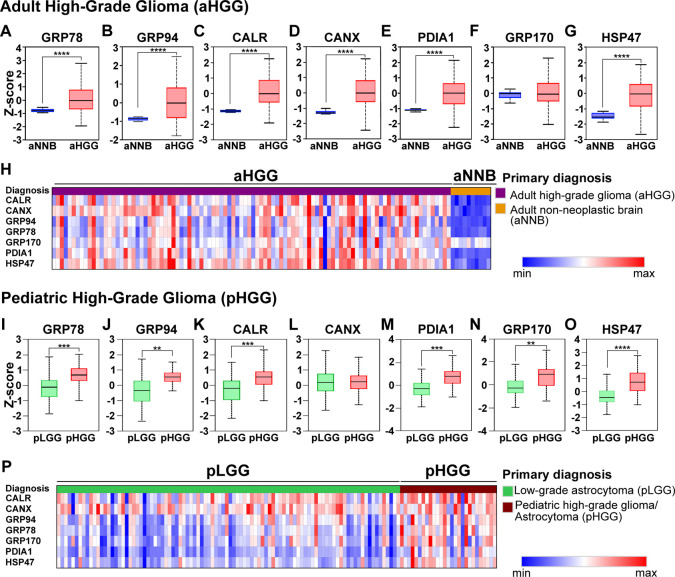
Relative protein expression of ER chaperones in glioma tumors and non-neoplastic brain tissues. Differential expression between adult HGG (aHGG; *n* = 99; shown in blue) and adult non-neoplastic brain (aNNB; *n* = 10; shown in red) for (A) GRP78, (B), GRP94, (C) CALR, (D) CANX, (E) PDIA1, (F) GRP170, and (G) HSP47. The z-scores were calculated to normalize the data. (H) Heatmap representing the mean normalized log_2_-ratio of ER chaperone protein expression levels in adult high-grade glioma (aHGG) and non-neoplastic brain (aNNB) samples. High expression is represented in red, while low expression is represented in blue. Differential expression between pediatric HGG (pHGG; *n* = 22; shown in red) and pediatric low-grade glioma (pLGG; *n* = 86; shown in green) is displayed for (I) GRP78, (J), GRP94, (K) CALR, (L) CANX, (M) PDIA1, (N) GRP170, and (O) HSP47. (P) Heatmap displaying the normalized ER chaperone protein expression levels across pLGG and pHGG samples. High expression is represented in red, and low expression is represented in blue. Data for aHGG were obtained from the “Clinical Proteomic Tumor Analysis Consortium (CPTAC) GBM Discovery Study” data set.^40^ Pediatric data were accessed from The Children Brain Tumor Tissue Consortium (CBTTC) data set.^39^ Expression difference between the groups was performed through the UALCAN data analysis portal.^42^ Box-whisker plots show the interquartile ranges (minimum, 1st quartile, median, 3rd quartile, and maximum). Significance was evaluated with a Welch’s test. **p < 0.01, ***p < 0.001, ****p < 0.0001. Heatmaps were generated through the PDC Common Data Analysis pipeline.^41^

Given the lack of publicly available pediatric proteome data sets which include NNB samples, pHGG and pLGG data were retrieved from the “Pediatric Brain Cancer Pilot Study” for the protein expression comparison.^39^ A significant (*p* < 0.01) overexpression in pHGG samples (*n* = 22) compared to pLGG (*n* = 86) was observed for all the ER chaperones, except CANX (Figure 3I–O). Also, the expression heatmap (Figure 3P) revealeda consistently lower expression of most chaperones in pLGG compared to pHGG, which displayed more heterogeneous expression and a tendency toward higher protein levels.

### 3.4 ER Chaperones Are Translocated to the Cell Surface of HGG Cell Lines

Increasing evidence supports intracellular protein relocalization to the cell membrane as a key feature of various cancers.^63^ ER chaperones are primarily kept in the ER by the presence of an ER retention signal (for instance, the amino acid sequence KDEL) in the C-terminus domain.^10^ Moreover, these chaperones lack transmembrane domains (except for CANX and GRP94; Figure 4) or GPI anchors (Table 2). However, the translocation of ER chaperones to other subcellular compartments has been increasingly observed.^19,54,55^ Previous reports have suggested the translocation of ER chaperones to the cell surface in various cancers.^12,64,65^ Nevertheless, membrane relocalization of ER chaperones in adult and pediatric gliomas, compared to normal tissue, is not elucidated. Identification of surface proteins is predominantly based on computational predictive tools. We investigated two computational-based surface protein databases, SURFY^66^ and The Cancer Surfaceome Atlas (TCSA),^67^ which reported the presence of only 1–2 ER chaperones in their samples (Figure 5A).^66^ However, these tools often overlook noncanonical membrane proteins (given their lack of transmembrane domains, membrane translocation signal peptide or GPI anchors or annotated ontologies).^11^ Therefore, we included in our analysis the Cell Surface Protein Atlas (CSPA), which used a glycocapture method for surface isolation and MS protein identification.^11^ In contrast to the prediction tools, the MS-based database detected the presence of 5 of these chaperones across 5 aHGG cell lines and 1 *ex-vivo* sample (Figure 5A).^11^ However, surfaceome profiling for pHGG samples and overall clinically relevant HGG samples is still lacking.

**Figure 4:**
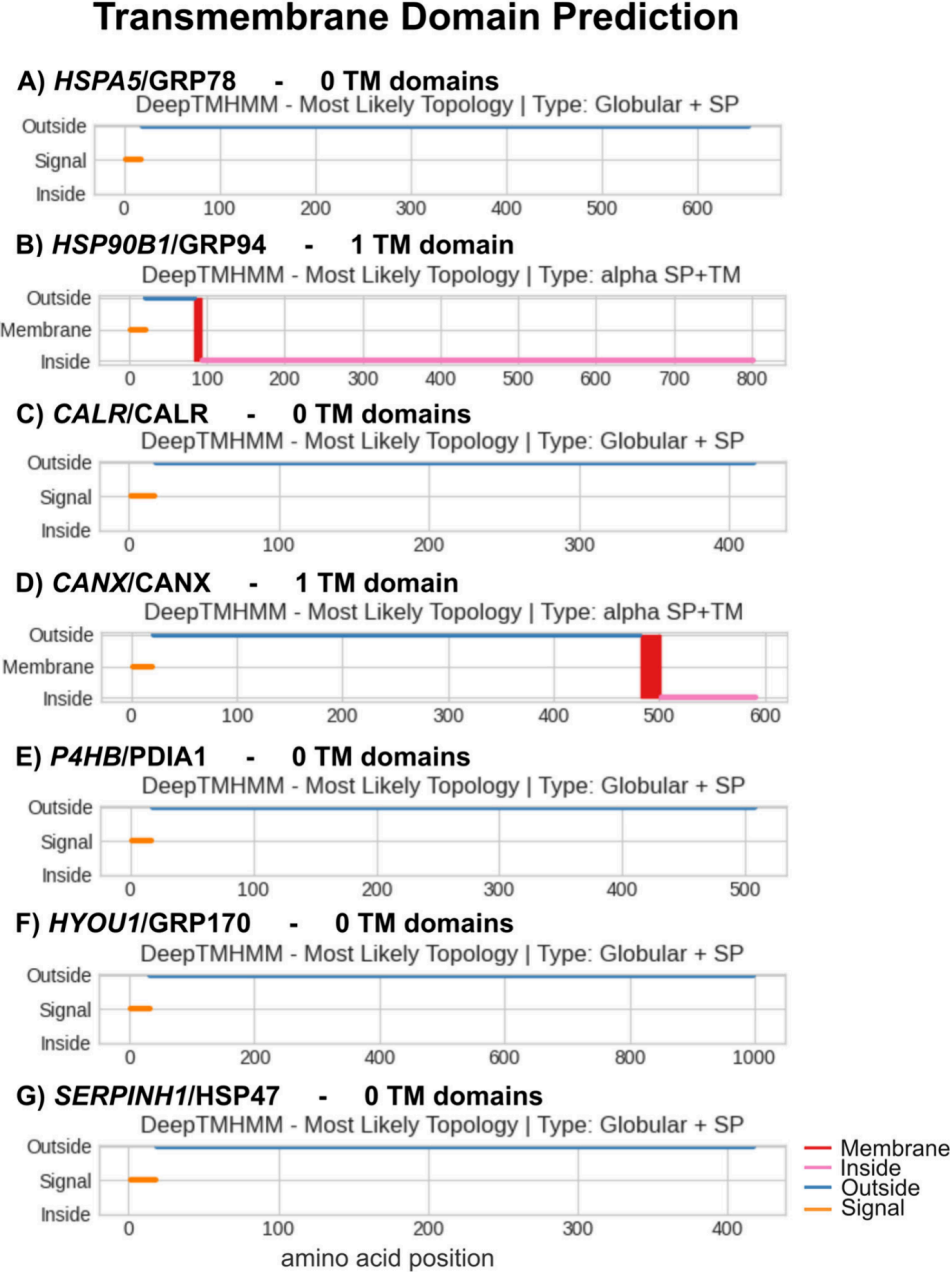
Prediction of the presence of transmembrane domains in 7 ER chaperones. The presence of transmembrane domains (TM) for each ER chaperone examined was predicted from the amino acid sequence, using the DeepTMHMM - 1.0 tool (DTU Health Tec).^45^ TM domains (“membrane”) represented in red; Intracellular domain (“Inside”) represented in pink; Extracellular domain (“outside”) represented in blue; Signal peptide (“signal”) represented in orange.

**Table 2:** Glycosylphosphatidylinositol Anchoring (GPI-anchor) Prediction in ER Chaperone Proteins

Protein	Prediction^a^	Probability
**GRP78**	No GPI anchor	0.995
**GRP94**	No GPI anchor	0.995
**CALR**	No GPI anchor	0.995
**CANX**	No GPI anchor	0.996
**PDIA1**	No GPI anchor	0.994
**GRP170**	No GPI anchor	0.994
**HSP47**	No GPI anchor	0.995

a Based on NETGPI 1.1.^44^

**Figure 5:**
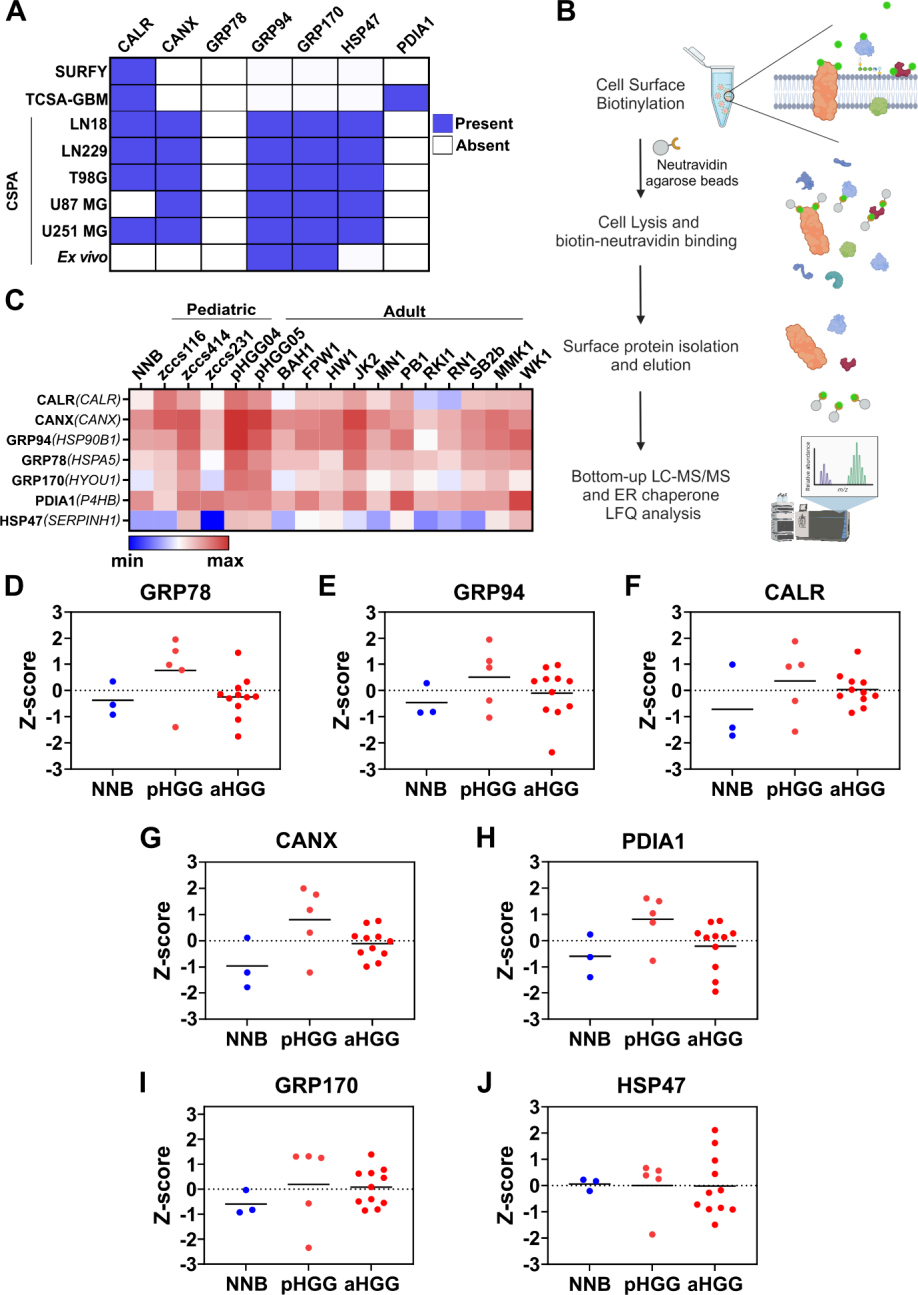
ER chaperones are translocated to the cell surface in brain cell lines. (A) Cell surface expression of ER chaperones based on data retrieved from the SURFY, The Cancer Cell Surface Atlas (TCSA-GBM) and the Cell Surface Protein Atlas (CSPA) databases. (B) Schematic representation of the workflow for cell surface protein biotinylation, isolation and identification workflow (created in BioRender). (C) Heatmap of the Log_2_-transformed intensities for ER chaperone surface expression in non-neoplastic brain (NNB) cell lines (*n* = 3), and each pHGG (*n* = 5) and aHGG (*n* = 11) cell lines. High expression is represented in red, and low expression is represented in blue. (D–J) ER chaperone surface expression (z-score) compared between NNB, pHGG and aHGG cells for (D) GRP78, (E) GRP94, (F) CALR, (G) CANX, (H) PDIA1, (I) GRP170, and (J) HSP47. Statistical significance was evaluated using a Kruskal–Wallis test with a Dunn’s posthoc test, using GraphPad Prism (v10.2.1). Horizontal line, mean value; dots, individual values. NNB, non-neoplastic brain; pHGG, pediatric high-grade glioma; aHGG, adult high-grade glioma.

Taking this into consideration, a collection of 11 aHGG PDCs, 3 pHGG PDCs, and 2 pHGG primary cell samples were obtained to assess the surface expression of the 7 ER chaperones of interest. The aHGG and pHGG cohort analyzed () demonstrated the heterogeneity of the disease (varying in characteristics *e.g.*, age, sex, and genomic differences). The surfaceome of the HGG samples was compared to human NNB cell models (astrocytes, brain pericytes and brain endothelial cells) to assess their cancer selectivity as surface targets. To evaluate the surfaceome, a cell surface protein biotinylation and isolation method was adapted (Figure 5B).^4,68^ Surface protein isolation was confirmed by Western blot in the established T98G glioblastoma cell line (). LC-MS/MS analysis and LFQ of the identified proteins was performed.^52^ Remarkably, the 7 ER chaperones of interest were identified in the surfaceome of all aHGG and pHGG PDC samples (Figure 5C), and in the non-neoplastic cells (Figure 5C), with >45% protein coverage (). A higher surface expression of GRP78, GRP94, CALR, CANX, or PDIA1 was observed in the pHGG cohort compared to the aHGG or NNB cohorts (Figure 5C,D–H). Furthermore, the differential expression analysis showed a trend for higher ER chaperone expression in pHGG and aHGG compared with NNB (Figure 5D–H), except for HSP47 (Figure 5J). No statistical differences were evident between the pHGG and aHGG cohorts.

### 3.5 Transcriptomic and Total Proteomic Analysis Does Not Reflect Surface Expression of ER Chaperone Proteins

Identifying new surface protein targets is of high relevance.^69^ It is well established that often mRNA levels do not correlate with total protein abundance.^70^ To explore how these differences relate to surface expression, we compared the mRNA and protein expression publicly available data (https://www.qimrb.edu.au/qcell)^33–35^ to the surface expression of ER chaperones in the 11 QIMR aHGG PDC analyzed herein. Accordingly, ER chaperone surface expression levels did not show the same expression pattern as its mRNA or total protein counterpart for all the ER chaperones examined or accross the different aHGG cells (Figure 6).^33–35^ For instance, *CALR* mRNA was highly detected in the HW1 sample, and showed low expression in JK2 sample (Figure 6), while the total protein expression was similar for both cell types, with slightly higher levels for JK2 than HW1 (Figure 6). In fact, at the surface, CALR showed one of the highest expressions in JK2, while HW1 showed a low abundance of CALR in this compartment (Figure 6). Similarly, in sample WK1, despite a low expression of *CALR* at the mRNA level, a high total CALR protein expression was observed, while the surface expression was slightly higher than the mean expression across the cell panel (Figure 6). An opposite trend was observed for GRP78/*HSPA5* in the BAH1 sample, which showed high expression at the mRNA and protein level compared to the other cells, but not for surface levels (Figure 6). Similarly, a weak nonsignificant Pearson correlation coefficient (*r* = 0.1–0.3) between mRNA and surface protein levels was found for all the ER chaperones examined, with CALR showing the greatest but still moderate correlation (Figure 7). Total vs. surface protein expression showed weak nonsignificant correlation for CANX (*r* = 0.23, *p* = 0.5), moderate correlation for CALR (*r* = 0.5, *p* = 0.1), and strong correlation (*r* = 0.58–0.88, *p* = 0.0003–0.064) for the rest of the chaperones (Figure 7). Therefore, these examples highlight the limited accuracy predicting surface availability of ER chaperones solely on mRNA or protein levels.

**Figure 6:**
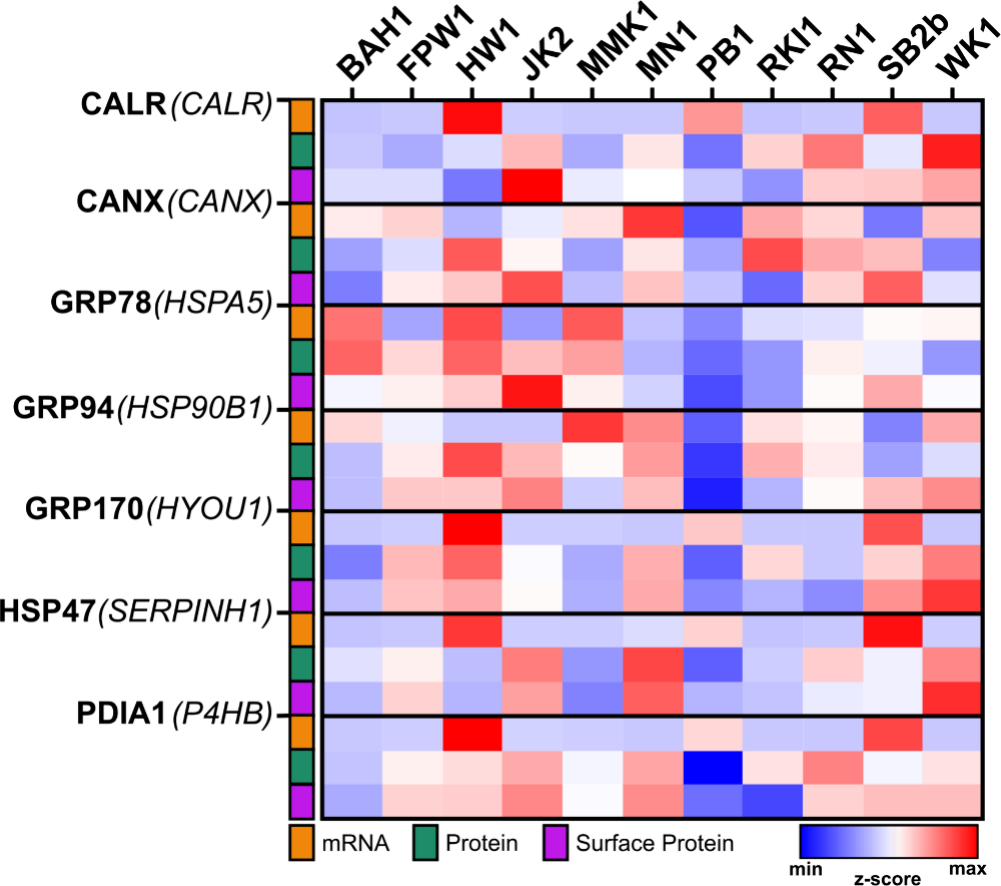
ER chaperone surface translocation levels are independent of mRNA of protein expression levels. Heatmap representing the ER chaperone expression (z-score) in the QIMR Q-cell adult glioblastoma PDC collection^33–35^ for mRNA (orange), total protein (green) and surface protein (purple). Levels of mRNA and total protein were obtained from publicly available data^33–35^ and surface protein were obtained by LC-MS/MS analysis. The z-score values were calculated per gene and protein across all samples. Heatmap was generated using GraphPad Prism (v10.2.1). Lower z-score values are represented in blue; high z-score values are represented in red.

**Figure 7:**
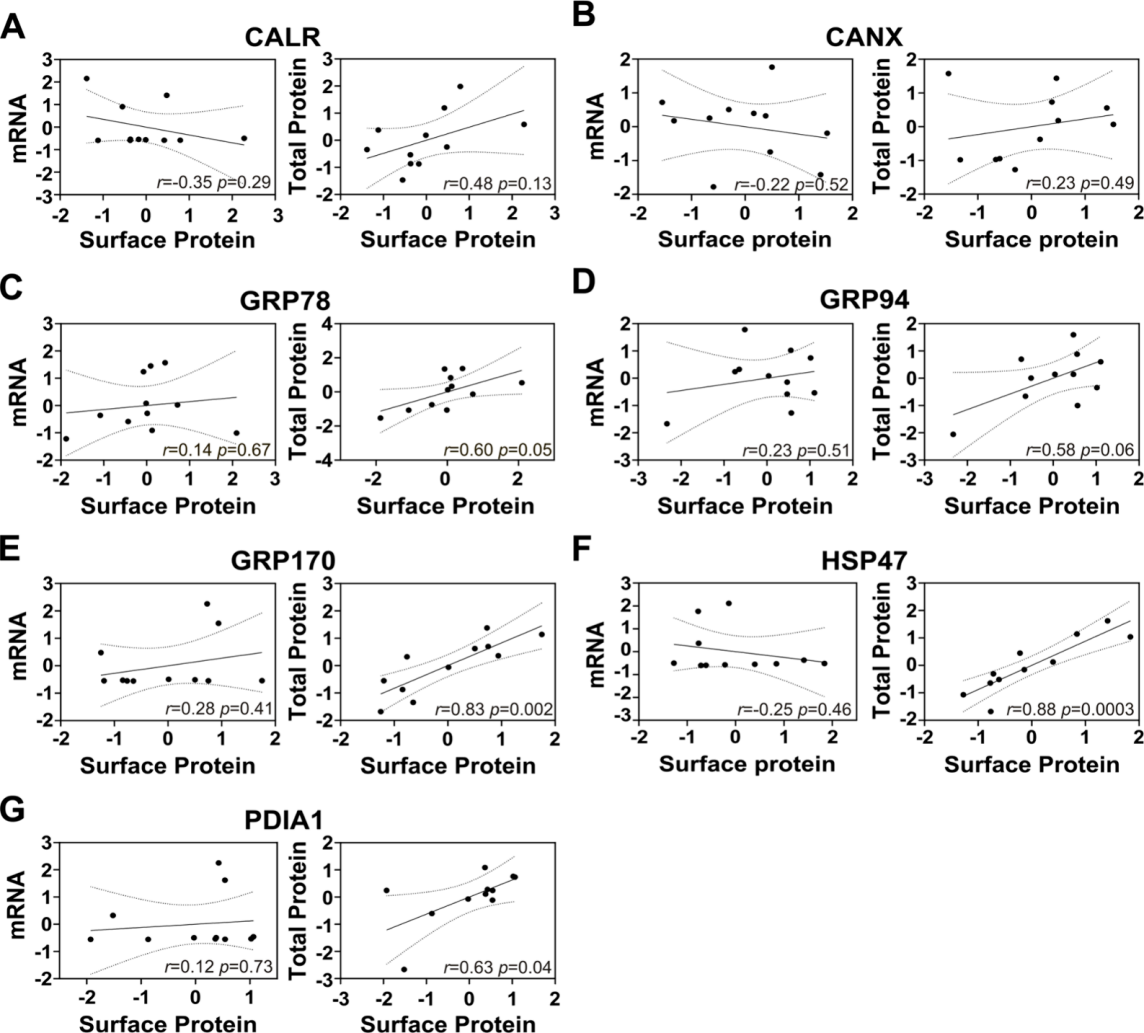
Pearson correlation coefficient between the mRNA or total protein and surface protein levels in aHGG PDCs. Expression levels for mRNA and total protein in the QIMR Q-cell adult glioblastoma PDC collection were obtained from publicly available data^33–35^ and surface protein were obtained by LC-MS/MS analysis. The z-score values were calculated per gene/protein across all samples. Pearson correlation coefficient (*r*) was calculated in GraphPad (v10.2.1). Solid line represents the best-fit linear regression; while the dotted line indicate the 95% confidence intervals.

## 4 Discussion

Elevated expression of ER chaperones in cancer relative to normal tissues has drawn attention to their potential as anticancer biomarkers and therapeutic targets.^8,9^ In fact, there is increasing evidence to support the relocalization of ER chaperone proteins to the cell membrane of tumor cells (*e.g.*, melanoma, colon, and gastric cancer, reviewed in refs 54, 55 ). Nevertheless, the expression of ER chaperones in aHGG and pHGG compared to nontumor samples has not been reported. Herein, we unraveled the expression landscape and identified the surface translocation of the ER chaperones GRP78, GRP94, PDIA1, CALR, CANX, HSP47, and GRP170 in aHGG and pHGG PDCs. Moreover, the transcriptomic and proteomic analyses confirmed a poor association between mRNA and the surface presence of these noncanonical membrane proteins. Importantly, this study highlights the need for surfaceome evaluation for each type of cancer without overlooking the surface expression of noncanonical membrane proteins.

Given the importance of transcriptomic and genomic predictions in identifying biomarkers and therapeutic targets, we examined the mRNA expression and mutational landscape of ER chaperones in HGG, and its correlation with patient survival. These analyses revealed a significant upregulation of ER chaperone mRNA expression in aHGG relative to aLGG and aNNB that was associated with tumor aggressiveness (Figure 1) and patient survival for *HSPA5*, *HSP90B1*, *P4HB*, and *SERPINH1* (Figure 2). Consistent with our findings, previous studies have demonstrated an association between ER stress pathway activation in glioblastoma patient tissues (*n* = 9) and high *HSPA5* and *HSP90B1* ER chaperone mRNA expression.^71^ Furthermore, a low mutational burden (<2.5%) in the genes of interest was identified, with no clinical implications to HGG found (). However, further investigation of post-translational modifications may reveal the presence of ER chaperone cancer-specific variants, as reported for GRP78 and GRP94 in gastric and breast cancer.^22,72^

On the other hand, pHGG samples exhibited a different trend, showing no evident association between ER chaperone mRNA expression and tumor aggressiveness (Figure 1) or overall survival (Figure 2). These findings are consistent with reports in other pediatric tumors, such as osteosarcomas, where ER chaperone mRNA levels remain unchanged^73^ and in acute myeloid leukemia, where *HSPA5* expression is downregulated compared to non-neoplastic cells.^74^ However, it is important to acknowledge the limitations of the pediatric data set as fifty-one percent (18/35) of the non-neoplastic samples (PsychENCODE data set) were derived from fetal brain tissue, which is known to express high levels of ER chaperones for proper neurodevelopment.^75^ Additionally, except for 1 sample with *P4HB* amplification, no gene mutations were found in the pHGG tissue samples (). In fact, genome-targeted strategies have poor success in brain tumors, particularly pediatric patients, partly due to the low mutational burden.^39^ Overall, these findings suggest that ER chaperones may represent promising biomarkers in aHGG but are likely less relevant in pHGG from the transcriptomic perspective.

Despite the differences observed between aHGG and pHGG at the transcriptomic level, both cancer types exhibited high total protein expression for most of the ER chaperones evaluated with multiple ER chaperones overexpressed across the majority of the samples (Figure 3). This highlights the known lack of correlation between total mRNA and protein levels.^62^ The upregulation of these chaperones may reflect activation of stress-response mechanisms of the tumor milieu that may contribute to tumor survival and therapeutic resistance.^19^ Notably, although ER chaperones are upregulated, this upregulation does not necessarily correlate with surface localization. Furthermore, the mRNA or total protein analysis herein does not discriminate between the intracellular (*e.g.*, cytoplasmic, ER) and surface protein localization.

The development of more selective therapies targeting membrane proteins requires a detailed knowledge of the cell surface composition. In fact, ER chaperones have been commonly overlooked in bioinformatic analyses and surface protein prediction tools due to their typical subcellular ER localization and lack of canonical membrane protein characteristics.^66,76,77^ Analysis of our MS data identified the 7 ER chaperones at the surface of aHGG and pHGG with no significant differences evident between these subgroups (Figure 5). Our analytical approach was not restricted to canonical membrane proteins but applied stringent quality control (>45% unique sequence coverage). The 7 ER chaperones exhibit a high degree of sequence similarity; however, our identification for each chaperone was based on unique peptide sequences, with no overlap between these chaperones (). Specifically, 45–78% of the peptide sequences detected for each chaperone were unique (). It should be noted that isoforms were not distinguished in this analysis, and future studies should further examine chaperone isoforms. Moreover, functional validation of the role of surface ER chaperones in gliomas is another avenue for future discovery.

Furthermore, while bioinformatic prediction tools can assist in determining protein localization based on specific amino acid sequence and structural features, such predictions often have limited accuracy, particularly in distinguishing noncanonical surface proteins, such as ER chaperones, which lack typical membrane protein characteristics (Figure 4, Table 2). The study herein did not examine the proportion of each chaperone translocated to the cell surface relative to the proportion of other subcellular compartments. Future studies could examine subcellular localization and assess isoform-specific peptides to determine whether specific chaperone peptide sequences were localized to the membrane or other specific cellular compartments.

Examination of public MS surfaceome raw data from aHGG cell lines using similar techniques confirmed the consistent identification of surface ER chaperones, which had often been neglected based on the data-filtering criteria favoring typical membrane protein selection.^78,79^ For instance, the surfaceome data published by Rose *et al.* (PRIDE data set identifier: PXD027110) exhibited the presence (in at least two of the three replicates) of CANX, GRP78, and GRP94 in U87 glioblastoma cells, NHC2 glioblastoma cells and human astrocyte cells.^78^ In this same data set, an upregulation of HSP47 was reported in these tumor cell lines relative to the human astrocytes.^78^ Moreover, PDIA1 was detected as a tumor-exclusive surfaceome protein in the NCH82 and U87 cell lines; however, it is flagged as “unspecific” surface protein by the CSPA database and was not further studied.^78^ Similarly, the data published by Ghosh *et al.* showed the presence of GRP78, GRP94, HSP47, and PDIA1 in three glioblastoma models (U87, T98G, and a glioma cancer stem cell) and normal neural stem cells. In this data set, cell surface CALR and CANX were identified as cancer-exclusive.^79^ However, the authors filtered the identified proteins by the presence of transmembrane domains, excluding CALR from their list of proteins of interest.^80^ Therefore, MS-based surfaceome analysis of different aHGG cell types aligns with our findings, despite these proteins being commonly disregarded as surface proteins. Additionally, specific ER chaperones, such as GRP78 and PDIA1, have been confirmed at the surface of aHGG cell lines by other groups using different techniques (*e.g.*, Western blot or flow cytometry).^81,82^ Notably, variability in surfaceome detection methods must be carefully considered, as experimental techniques can influence the detection and quantification of cell surface proteins.^83^ Standardization across studies is essential to compare findings and define robust therapeutic targets.^11,84^ In fact, it remains crucial to validate all MS findings using other techniques, such as flow cytometry.

It is important to acknowledge that the relatively small pediatric cohort sizes analyzed across the figures herein may reduce the statistical power and obscure subtle expression differences. This limitation is particularly relevant when interpreting marginal findings such as *CALR* mRNA downregulation in pHGG. Validation in larger pediatric cohorts will be essential as they become available to confirm and strengthen our multiomics observations. Additionally, it is important to note that our mRNA and protein data sets come from multiple repositories, each produced by different research groups. We would like to acknowledge this limitation and that multiomics correlations could be limited due to inconsistencies between data sources.

Moreover, post-translational modifications of ER chaperones are known to modulate their localization and function. For example, acetylation of the ER chaperone GRP78 modifies its secretion in colon cancer cells and its translocation to the membrane in cholangiocarcinoma.^85,86^ Given the importance of post-translational modification in influencing chaperone activity and therefore cell survival and death, it is crucial that future studies examine the post-translational modification of ER chaperones, cancer-specificity, functional consequences and whether this could impact cellular localization.^10,18^

Notably, these noncanonical membrane proteins were detected at the surface of non-neoplastic brain cell lines (human astrocytes, pericytes, and endothelial cells) herein, demonstrating their lack of selectivity to cancer. This limitation suggests that surface ER chaperones may have restricted potential as drug targets or biomarkers in HGG, in contrast to studies of prostate cancer and breast carcinoma (tested in mouse models)^87^ or hepatic, and lung cancer cells (tested *in vitro*).^88^ Therefore, a cancer- and cell-type-specific surfaceome evaluation is needed to confidently determine the presence of a target in this subcellular compartment. Notably, ER chaperones were not overexpressed at the cell surface in both aHGG and pHGG compared to NNB. Nevertheless, overall total ER chaperone expression was upregulated in aHGG relative to NNB and pHGG relative to pLGG, suggesting heightened activation of the UPR. This likely reflects a cellular stress adaptation driven by the tumor microenvironment and oncogenic signaling and highlights the lack of correlation between surface expression, and total expression or UPR activation.^62,89,90^ Importantly, gliomas are an extremely heterogeneous cancer type and it is therefore likely that the surfaceome is also patient dependent.^91^ Even more, the overall expression results of the ER chaperone family in HGG highlight the importance of an integrative analysis, with a broader surface protein identification strategy, not restricted to canonical membrane proteins. Current membrane protein prediction tools coupled with transcriptomic and overall proteomic analyses are not predictive of surface protein expression levels. This was clearly observed in the aHGG cohort analyzed for the 7 ER chaperones evaluated (Figure 6, Figure 7).

## 5 Conclusion

Herein, we examined the differences between mRNA, total protein, and surface expression levels of seven ER chaperones in HGG. Our findings highlight the importance of an integrative expression analysis in the finding of new biomarkers and therapeutic targets. The 7 ER chaperones investigated showed different mRNA expression profiles between aHGG, pHGG and NNB samples. The total protein expression was significantly higher for the 7 ER chaperones in aHGG and pHGG compared to NNB samples. Furthermore, the translocation of 7 noncanonical surface proteins, namely, ER chaperones, was detected by MS across a comprehensive panel of 16 PDCs or primary cells, including adult and pediatric HGG samples. There was a lack of cancer-exclusivity observed for the ER chaperone surface expression with expression evident in 3 non-neoplastic cell lines. Moreover, mRNA expression showed a poor correlation to total protein and surface protein expression. Therefore, understanding the transcription and translation expression profile along with protein localization is key to unraveling cancer selective therapeutic targets. Further study and identification of noncanonical surface proteins could provide prompt targets for biomarker or immunotherapy design (*e.g.*, monoclonal antibodies, CAR T-cells, antibody-conjugated nanomedicine) in adult and pediatric HGG.

## Supplementary Material





## Data Availability

The aHGG mRNA expression, survival analysis and mutation analysis were generated from publicly available TCGA Research Network Glioblastoma and low-grade glioma data sets. Data can be accessed directly through TCGA (https://www.cancer.gov/tcga) or the servers specified in materials and methods section (XENA browser (mRNA expression; https://xenabrowser.net/), R2 genomics (survival analysis; https://r2.amc.nl/) or cBioPortal (mutational landscape; https://www.cbioportal.org/)). The pHGG mRNA data set can be accessed through the StJude Cloud (https://www.stjude.cloud/) upon approval. Pediatric non-neoplastic data were accessed through the publicly available data from the PsychEncode Consortium. Proteomic data are available at the Clinical Proteomic Tumor Analysis Consortium (PDC Study ID pHGG: PDC000180 and aHGG: PDC000204; https://proteomics.cancer.gov/programs/cptac). Qcell mRNA and proteomic profile data for the 10 aHGG PDC cells was obtained from their webpage (https://www.qimrb.edu.au/qcell). The mass spectrometry surfaceome data for aHGG, pHGG and NNB has been deposited to the ProteomeXChange Data set Identifier: PXD015545.^53,92^

## Notes

Figure S1. Comparison of clinical attributes and mutational status of ER chaperone genes in HGG patients. Figure S2. Relative protein expression of ER chaperones in glioma tumors and non-neoplastic brain tissues. Figure S3. Isolation of the surface surface protein EGFR by biotinylation and neutravidin pulldown. Table S1. Characteristics of adult high-grade glioma patient-derived cell samples. Table S2. Characteristics of the pediatric high-grade glioma patient-derived cell samples. (PDF)
Table S3 Log2-transformed LFQ ER chaperone data in aHGG, pHGG and NNB. Table S4 ER chaperone peptides identified at the surface of aHGG, pHGG and NNB. (XLSX)

## Abbreviations

CNS: Central nervous system
ER: endoplasmic reticulum
NNB: non-neoplastic brain
HGG: high-grade glioma
pHGG: pediatric high-grade glioma
aHGG: adult high-grade glioma
LGG: low-grade glioma
MS: Mass spectrometry
LFQ: Label-free quantification
