# Supplementary material for: Surface Proteomic Analysis Reveals the Presence of Noncanonical Cell Membrane Endoplasmic Reticulum Chaperones in High-Grade Gliomas
Source: J Proteome Res. 2025 Nov 25;25(1):169–84. doi: 10.1021/acs.jproteome.5c00616 (PMC12772127; doi:10.1021/acs.jproteome.5c00616)
Supplement: Supplementary file 1 [file pr5c00616_si_008.pdf]

SUPPORTING INFORMATION (SI1)

Surface proteomic analysis reveals the presence of non-canonical cell membrane endoplasmic reticulum chaperones in high-grade gliomas.

Alexis Z Minchaca[1,2,3,4], Jean Bertoldo[1,2], Philipp Graber[1,2,3,4], Dong-Hun Bae[1,2], Nisitha Jayatilleke[1], Chelsea Mayoh[1,2], Brett W Stringer [6], Louise Ludlow [7,8,9], Maria Kavallaris [1,2,4,5], Angelica M Merlot*[1,2,3,4,5]

1. Children's Cancer Institute Australia, Lowy Cancer Research Centre, UNSW Sydney, NSW 2052, Australia

2. School of Clinical Medicine, Faculty of Medicine and Health, UNSW Sydney, NSW 2052, Australia

3. UNSW Centre for Childhood Cancer Research, Faculty of Medicine & Health, University of New South Wales, Kensington, NSW 2031, Australia

4. UNSW Australian Centre of Nanomedicine, Faculty of Engineering, UNSW Sydney, NSW 2052, Australia

5. UNSW RNA Institute, Faculty of Science, UNSW, Sydney, NSW 2052, Australia.

6. Institute for Biomedicine and Glycomics, Griffith University, Brisbane, QLD 4111, Australia.

7. Murdoch Children's Research Institute, Parkville, VIC 3053, Australia

8. Children's Cancer Centre, The Royal Children's Hospital, Parkville, VIC 3053, Australia

9. Department of Paediatrics, The University of Melbourne, Parkville, VIC 3052, Australia

*Correspondence to: a.merlot@unsw.edu.au

Table of contents

Page 3 Supplementary Figure S1. Comparison of clinical attributes and mutational status of ER chaperone genes in HGG patients.

Page 4 Supplementary Figure S2. Relative protein expression of ER chaperones in glioma tumors and non-neoplastic brain tissues.

Page 5 Supplementary Figure S3. Isolation of the surface surface protein EGFR by biotinylation and neutravidin pulldown.

Page 6 Supplementary Table S1. Characteristics of adult high-grade glioma patient-derived cell samples

Page 7 Supplementary Table S2. Characteristics of the pediatric high-grade glioma patient-derived cell samples

Page 8 Supplementary Table S3 Log2-transformed LFQ ER chaperone data in aHGG, pHGG and NNB (excel file)

Page 8 Supplementary Table S4 ER chaperone peptides identified at the surface of aHGG, pHGG and NNB (excel file)

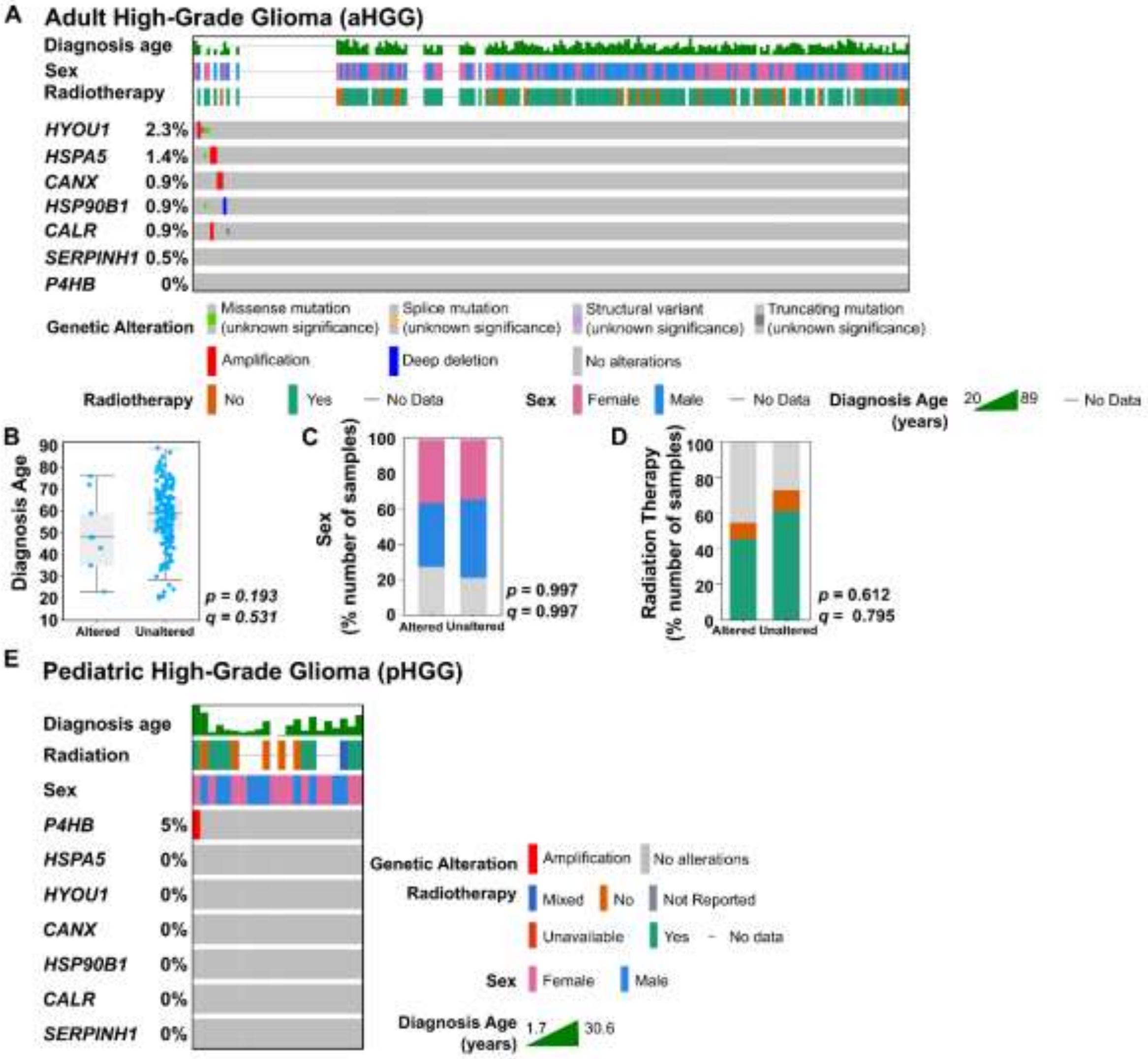

Supplementary Figure S1. Comparison of clinical attributes and mutational status of ER chaperone genes in HGG patients. *(A) Oncoplot summarizing the distribution of genomic alterations of the ER chaperone genes per sample in aHGG patients (n=221) from the TCGA-GBM PanCancer cohort (reclassified according to the latest WHO CNS5 classification based on 26). Correlation analysis of (**B**) diagnosis age, (**C**) sex and (**D**) radiotherapy between the mutated and unaltered samples. Wilcoxon test (**B**) or chi-square test (**C** and **D**) analysis was performed. (**E**) Oncoplot summarizing the distribution of genomic alterations of ER chaperones genes in the pHGG cohort (n=22) obtained from the "Pediatric Brain Cancer (Clinical Proteomic Tumor Analysis Consortium, CPTAC/CHOP)" dataset[39]. The oncoplots, and graphs were created through cBioPortal (https://www.cbioportal.org).[39]*

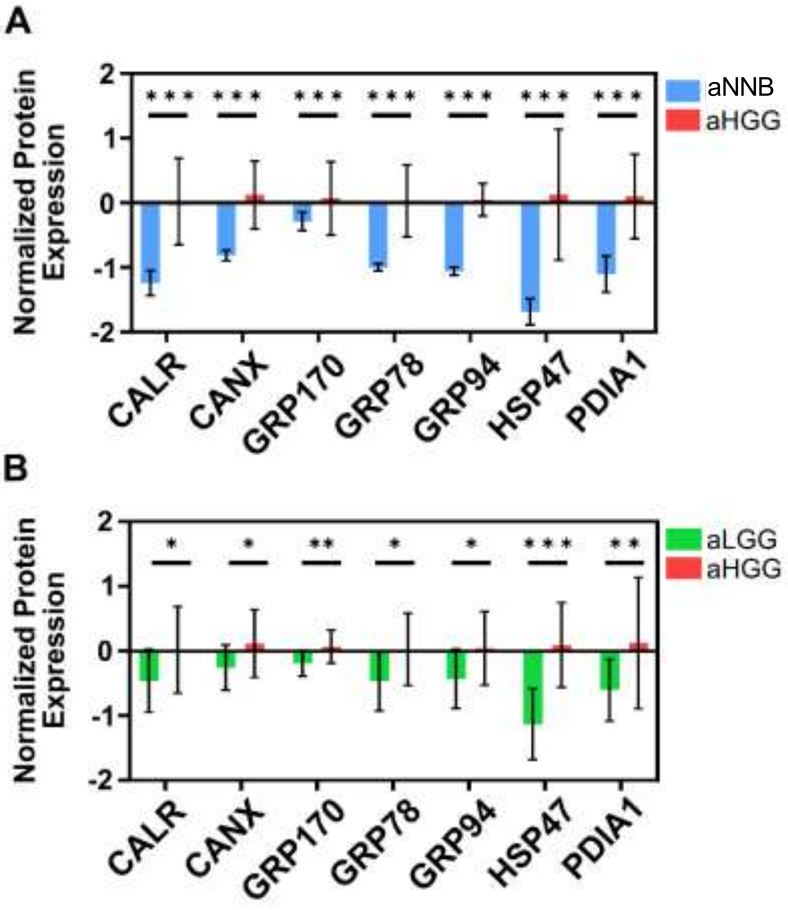

Supplementary Figure S2. Relative protein expression of ER chaperones in glioma tumors and non-neoplastic brain tissues. *Proteomic data (normalized to log_2 sample intensity/global internal standard control) from each adult NBB (n=4), LGG (n=9) and HGG (n=39) patient were retrieved (PXD015545)[43] and ER chaperone expression was compared between (**A**) NNB and aHGG or (**B**) aLGG* and *aHGG. Significance was determined using an unpaired t-test, in GraphPad Prism (v10.2.1). Bar, mean value; Error bar, SD. For both graphs, *, p<0.05; **, p<0.01; ***, p<0.001; ****, p<0.0001.*

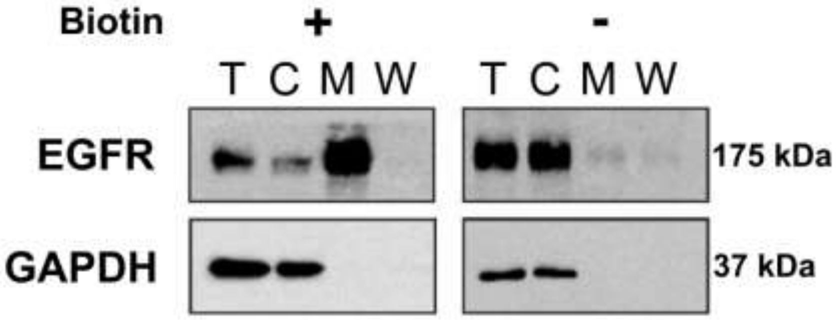

Supplementary Figure S3. Isolation of the surface protein EGFR by biotinylation and neutravidin pulldown. *Immunoblot showing the enrichment of the epidermal growth factor receptor (EGFR) in the membrane fraction (M) of T98G cells after biotin labelling. Glyceraldehyde 3-phosphate dehydrogenase (GAPDH) was used as intracellular/cytoplasmic control (C). The whole cell lysate (T) was loaded as a control of total protein expression. The wash fraction (W) was included to confirm negligible protein loss. Approximately, 1mg of total protein was loaded into the neutravidin resin post-labelling. From the membrane protein eluate (M), 30 µL were loaded into a 10% polyacrylamide gel, and 10% of that volume was loaded in the gel for the T, C and W fractions.*

Supplementary Table S1. Characteristics of adult high-grade glioma patient-derived cell samples

Sample ID	Age (years)	Sex	Tumor Type	Glioblastoma type[a]	MGMT methylation	Mutation	Amp/Del
**BAH1**	75	Female	Primary	CL	Yes	*EGFRvIII*<br>*PTEN V133I*	*EGFR*<br>*PIK3R1del*
**FPW1**	68	Male	Primary	Mes/CL	No	*PTEN R130Q*	N/A
**HW1**	54	Female	Primary	MES	Yes	*EGFR A289V*	N/A
**JK2**	75	Male	Primary	MES	No	*TP53 R110L*	*PTENdel*
**MMK1**	80	Female	Primary	MES	No	*PTEN F56V*<br>*IDH1 Y183C*	N/A
**MN1**	84	Female	Primary	MES	No	*MET E168D*<br>*PIK3C2G P129T*	
**PB1**	57	Male	Primary	PN/CL	No	*PTEN R130**<br>*PIK3C2G X1446S*	
**RKI1**	57	Female	Primary	MES	Yes	*ATRX D808G*<br>*SETD2 R472H*	*MYC*
**RN1**	56	Male	Primary	MES	No	*MET T992I*	*EGFR* gain
**SB2b**	48	Male	Recurrent	MES/CL	Yes	*EGFR A289V, H304Y*<br>*MET C800F*<br>*IDH1 V178IIK*<br>*PIK3CA Y1021H*<br>*PIK3C2G A2T*<br>*MMD2 W329C*<br>*SERD2 T451A*	*PTEN*
**WK1**	72	Male	Primary	CL	No	*PIK3CA H1047Y*<br>*SETD2 E670K*	*PTEN*

Supplementary Table S2. Characteristics of the pediatric high-grade glioma patient-derived cell samples

Sample ID	Age (years)	Sex	Tumor Type	Diagnosis	Mutations	Amplifications
**zccs116**	11	Male	Primary	HGG hemispheric	*BRAFV600E*<br>*CDKN2A/B* loss; germline *APCmut*	
**zccs414**	19	Male	Primary	HGG hemispheric	*TP53*, *RB1*, *NF1*, *PTEN*, *MSH6*; germline *MSH2*	*C19MC*
**zccs231**	11	Female	Recurrent	HGG hemispheric	N/A	N/A
**pHGG04**	7	Female	Primary	HGG	N/A	N/A
**pHGG05**	8	Male	Primary	HGG	*H3 G34R*<br>*TP53 R175H*	

Supplementary Table S3. Log_2-transformed LFQ ER chaperone data in aHGG, pHGG and NNB

- Excel file

Supplementary Table S4. ER chaperone peptides identified at the surface of aHGG, pHGG and NNB

- Excel file
